# The Impact of Malnutrition on Post-Operative Complications in Patients with Ovarian Cancer: A NSQIP Study

**DOI:** 10.3390/curroncol33060358

**Published:** 2026-06-15

**Authors:** Lina Salman, Anjali Kulkarni, Jacob McGee

**Affiliations:** 1 Division of Gynecologic Oncology, Durham Regional Cancer Centre, Lakeridge Health, Oshawa, ON L1G 2B9, Canada; 2 Department of Obstetrics and Gynecology, Queen’s University, Kingston, ON K7L 3N6, Canada; 3 Division of Gynecologic Oncology, London Health Sciences Centre, University of Western Ontario, London, ON N6A 5A5, Canada; 4 Division of Gynecologic Oncology, Nova Scotia Cancer Centre, Dalhousie University, Halifax, NS B3H 1V7, Canada; 5 Department of Biostats and Epidemiology, University of Western Ontario, London, ON N6A 3K7, Canada

**Keywords:** ovarian cancer, malnutrition, surgery, complication

## Abstract

We aimed to evaluate the impact of malnutrition on post-operative mortality and complications in patients undergoing surgery for ovarian cancer (OC). In this retrospective cohort study utilizing the ACS NSQIP database (2013-2022), all individuals with a diagnosis of OC were included. Patients were classified as having “malnutrition” if they met any of the following: (1) pre-operative albumin level <3.5 g/dL; (2) ≥10% weight loss over 6 months + BMI = 18.5–20 kg/m^2^ in patients <70 years old or BMI = 18.5–22 kg/m^2^ in patients ≥70 years); and (3) BMI < 18.5. Baseline characteristics, 30-day post-operative mortality and complications were compared between “malnutrition” and “no malnutrition” groups. Of the 20,174 included, 8744 (43.3%) had malnutrition and 11,430 (56.7%) had no malnutrition. The malnutrition group had longer total length of hospital-stay (mean days 4.78 vs. 4.17, *p* < 0.0001), higher rates of venous thromboembolism, and higher cardiac morbidity compared to “no malnutrition”. On univariate analysis, mortality was higher in the “malnutrition” group (0.8% vs. 0.3%, *p* < 0.0001). This remained significant after adjusting for potential confounders (aOR 2.92, 95% CI 1.92–4.43, *p* < 0.0001). In conclusion, in patients undergoing surgery for OC, malnutrition increased the risk of post-operative mortality and complications. Malnutrition assessment should be integrated in pre-operative counseling in patients undergoing surgery for OC.

## Simple Summary

Malnutrition is common among patients with ovarian cancer. In this study, we evaluated the impact of malnutrition on post-operative complications in patients with ovarian cancer, using the American College of Surgeons-National Surgical Quality Improvement Program. We found that malnutrition was associated with increased 30-day post-operative morbidity including infections, cardiac morbidity and venous thromboembolism. There was also an increased post-operative mortality associated with malnutrition. Our findings suggest integrating malnutrition assessment in routine practice in patients undergoing surgery for ovarian cancer. Nevertheless, future studies are needed to assess the role of nutritional intervention in decreasing post-operative morbidity and mortality in these patients.

## 1. Introduction

In Canada, approximately 3000 new cases of ovarian cancer are diagnosed yearly, with 70–75% of cases presenting with advanced disease at presentation due to its association with non-specific symptoms [1]. Intra-peritoneal dissemination is common in advanced ovarian cancer, and it is associated with nausea, lack of appetite, dyspepsia and abdominal pain [2]. These symptoms can lead to poor oral intake and based on previous reports, up to 70% of patients with ovarian cancer suffer from malnutrition upon diagnosis [3,4].

Different assessment methods for determining nutritional status in cancer patients have been studied and validated [3,4]. The European Society for Clinical Nutrition and Metabolism (ESPEN) has established criteria to define malnutrition [5]. This includes body mass index (BMI) < 18.5 kg/m^2^, or combined weight loss with reduced BMI. These criteria have previously validated and used in previous studies to define malnutrition [6]. Serum albumin levels are used as an indicator for malnutrition, given that the liver’s production of albumin is decreased when energy stores are low [7].

Malnutrition has been shown to increase risk for hospitalization and surgical complications in the general population as well as in patients with cancer [8,9,10]. Moreover, pre-operative nutritional supplements have been shown to decrease post-operative complications, mainly infectious morbidity [11]. For gynecologic cancers, previous studies found that patients with malnutrition are at increased risk for post-operative complications [6,12]. Therefore, multiple international guidelines recommend incorporating pre-operative malnutrition assessment in patients with gynecologic cancer [13,14,15], and suggest providing nutritional support for patients found to have malnutrition.

While there is evidence that malnutrition is associated with higher risk of peri-operative complications among patients with gynecologic cancer, paucity of data exists on the association between baseline nutritional status and peri-operative complications including mortality in patients with ovarian cancer specifically.

In this study, we aimed to determine whether malnutrition is an independent risk factor for 30-day post-operative complications in patients undergoing surgery for ovarian cancer.

## 2. Materials and Methods

### 2.1. Database and Study Population

This is a retrospective cohort study using the American College of Surgeons-National Surgical Quality Improvement Program (ACS-NSQIP) Participant Use Data File (PUF). ACS-NSQIP is a prospective, multicenter, validated registry in the United States and Canada, including data from over 600 centers. While participating centers are mainly from the United States and Canada, there are several international sites that are part of the registry. Individuals included in this database are deidentified. ACS-NSQIP collects peri-operative data and 30-day post-operative complications by certified data abstractors. Data quality assurance of ACS-NSQIP is performed through a routine auditing process at each participating site [16]. A detailed list of variables collected in the ACS NSQIP Participant Use File (PUF) is available in the official user guide published by the American College of Surgeons [17].

For this study, we included all patients who underwent surgery for ovarian cancer between the years 2013–2022, using International Classification Disease Codes (ICD), 9th revision (ICD-9 codes: 183.0, 183.2, 183.8, 183.9, 236.2), or 10th revision (ICD-10 codes: 56.0, 56.1, 56.2, 56.9). Patients <18 years or those who received Hyperthermic Intraepithelial Chemotherapy (HIPEC), are excluded from NSQIP-PUF data registry.

The study was approved by the Institutional Review Board approval of Western University (ID 125548). Informed consent was waived due to the study design and deidentified nature of the database.

### 2.2. Outcomes and Covariates

The primary outcome of this study was to compare 30-day post-operative complications and mortality between patients undergoing surgery for ovarian cancer and with malnutrition to those with no malnutrition.

Definition of malnutrition was based on pre-operative albumin levels, ESPEN 1, and ESPEN 2 criteria [5]. Patients were classified as having “malnutrition” if they met any of the following criteria:

(1) Pre-operative albumin level <3.5 g/dL;
(2) BMI < 18.5 (ESPEN 1 criteria);
(3) ESPEN 2 criteria (≥10% weight loss over 6 months + BMI = 18.5–20 kg/m^2^ in patients <70 years old or BMI = 18.5–22 kg/m^2^ in patients ≥70 years old).

Disseminated cancer is defined by NSQIP as cancer that has: (1) spread to one or more sites in addition to the primary site, and (2) presence of multiple metastases indicating the cancer is widespread, fulminant, or near terminal [17]. Frailty was defined using the 5-item modified frailty index (mFI-5) with one point assigned to each of the following variables: partially/totally dependent functional status, diabetes, hypertension requiring medications, chronic obstructive pulmonary disease and congestive heart failure [18]. Participants with mFI-5 ≥2 were considered “frail” based on findings from previous publications [19,20].

### 2.3. Statistical Analysis

Descriptive statistics were used to compare characteristics between patients with malnutrition and patients with no malnutrition. Univariate analysis using two-tailed Student *t*-test for continuous variables and Chi-squared test for categorical variables were applied and results reported with odds ratios (OR). Multivariate logistic regression was used to determine factors associated with 30-day post-operative mortality, with adjusted odds ratios (aOR) reported, controlling for confounders. Potential confounders entered in the model were age ≥ 70 years old, smoking, frailty, non-elective surgeries, and American Society of Anesthetiologists score (ASA) ≥3. Collinearity diagnostics confirmed that frailty and ASA were not collinear. In this analysis, we included the selected confounders based on clinical significance and known impact on mortality. Backward elimination was used to determine variables to remain in the model and smoking was the only variable removed when assessing the association between malnutrition and death. Other outcomes that were significant on univariate analysis were assessed using logistic regression model, and these included: (1) Venous thromboembolism (VTE): if any event of deep venous thrombosis (DVT) or pulmonary embolism (PE); (2) “Cardiac morbidity”: if any event of post-operative myocardial infarction or cariad arrest; and (3) Infectious morbidity: if any event of deep surgical site infection, pneumonia, sepsis, or septic shock. For VTE, frailty and high ASA remained in the model. For cardiac morbidity, elderly, frailty and high ASA remained in the model. While for infectious morbidity, all variables retained in the model. Value of <0.05 was considered statistically significant. Statistical analysis was performed using SAS OnDemand (Version 9.4, SAS Institute Inc., Cary, NC, USA).

## 3. Results

Of the 20,174 included in the study, 8744 (43.3%) had malnutrition and 11,430 (56.7%) had no malnutrition. The “malnutrition” group was slightly younger (mean age 58.6 vs. 59.8, years old, *p* < 0.0001) and had higher rates of smoking (13.5% vs. 11.8%, *p* < 0.0005). The malnutrition group had higher rates of ascites (17.5% vs. 12.5%, *p* < 0.0001), but lower rates of disseminated cancer (26.6% vs. 33.6%, *p* < 0.0001), compared to the “no malnutrition” group, as shown in Table 1. There was no significant difference between the groups with respect to BMI.

The “malnutrition” group had shorter operative time (176.5 vs. 194.1 min, *p* < 0.0001) and longer total length of hospital-stay (mean days 4.78 vs. 4.17, *p* < 0.0001). There was no difference in “non-home” discharge destination between groups (17.2% vs. 17.7%, *p* = 0.36), and total units of transfusion (2.47 vs. 2.32, *p =* 0.424), Table 2.

As for post-operative complications, “malnutrition” had higher rates of deep surgical site infection (0.5% vs. 0.3%, *p* = 0.034), septic shock (0.9% vs. 0.5%, *p* = 0.001), and pneumonia (1.9% vs. 1.4%, *p* = 0.0001). In addition, they had higher cardiac morbidity compared to “no malnutrition”, Table 3. Events of DVT and PE were higher in the malnutrition group. Although 30-day mortality rate was low, it was higher in the “malnutrition” group (0.8% vs. 0.3%, OR 2.99, *p* < 0.0001).

On multivariate logistic regression, malnutrition remained significantly associated with 30-day post-operative mortality (aOR 2.92, 95% CI 1.92–4.43, *p* < 0.0001), VTE (aOR 1.32, 95% CI 1.08–1.60, *p* = 0.005), infectious morbidity (aOR 1.75, 95% CI 1.42–2.17, *p* < 0.0001) and cardiac morbidity (aOR 2.19, 95% CI 1.52–3.17, *p* < 0.0001).

## 4. Discussion

In this large retrospective cohort study, patients with malnutrition undergoing surgery for ovarian cancer had higher rates of post-operative complications compared to patients with good nutritional status. These complications include VTE, infectious morbidity and cardiac morbidity. In addition, malnutrition was associated with 3-fold increase in the odds of death within 30-day post-operatively.

We found higher rates of DVT and PE in patients with malnutrition. There is limited published data on the direct association of malnutrition and VTE in cancer patients. Previous studies have shown that low levels of vitamin B6, which can result from malnutrition, is associated with increased risk of VTE [21]. While this could provide partial explanation for our findings, there are other factors that can contribute to an increased risk of post-operative VTE such as the extent of surgery, burden of disease and patient’s mobility post-operatively. In our cohort, patients with malnutrition did have longer hospital stays post-operatively, which could also contribute to increased VTE risk.

In this study, malnutrition was associated with higher rates of post-operative infections including surgical site infection, pneumonia and sepsis. Malnutrition has been linked to increased risk of infection through impaired immune system [22]. One studied pathway is nutrient deficiencies leading to low lymphocyte levels, which is potentially a risk factor for infections. In fact, there is emerging data showing that immunonutrition can decrease the risk of post-operative infection, and can be considered in patients with gynecologic cancer [23]. A prospective cohort study from Pergialiotis et al. showed that malnutrition is associated with increased post-operative infections in patients undergoing surgery for gynecologic cancer [24].

While these findings support our results, they included all types of gynecologic cancer and only 37% of the study cohort had ovarian cancer.

Although the absolute risk of post-operative cardiac morbidity is low, previous studies have shown that the risk is increased after urgent surgeries [25]. In our study, rates of post-operative cardiac morbidity were higher in the group of patients with malnutrition, even when adjusting for non-elective surgeries. Data on the direct association between malnutrition and cardiac morbidity following non-cardiac surgeries remain limited. However, several studies have demonstrated that malnutrition is significantly associated with an increased risk of major cardiac events after cardiac surgeries [26].

Lastly, in our cohort people with malnutrition had higher risk of post-operative mortality. This has been consistent over several studies [27,28]. A systematic review by Brown et al. has shown that malnutrition is associated with decreased survival in cancer patients undergoing surgery [29]. The main types of cancers included in this review were gastrointestinal cancers, and representation of gynecologic cancers was minimal.

Interestingly, Patients with malnutrition had shorter operative times; however, despite this, they experienced higher rates of postoperative complications, contrary to the expected association between longer operative duration and adverse outcomes [30].

The strength of this study is that it is a multi-centre large retrospective study. The data from ACS-NSQIP is known to be highly accurate and reliable which further supports our results and conclusions [16]. Nonetheless, this study is not free of limitations. The database does not include indication for the procedure and whether patients received chemotherapy prior to surgery. Indications for surgery vary and can include primary cytoreduction, interval cytoreduction, or urgent surgery such as in case of a bowel obstruction. Different indications and the extent of surgery could affect the rate of post-operative complications. We tried to mitigate the effect of urgent procedures by adjusting for it as a confounder. While ACS-NSQIP database does not include stage of disease, the variable “disseminated cancer” as defined in the methods section, can indirectly represent disease stage. Interestingly, in this cohort, patients without malnutrition exhibited higher rates of disseminated cancer compared with those classified as malnourished. This unexpected pattern raises concerns about the validity of this variable, as patients with disseminated disease would typically be expected to have a higher risk of malnutrition, given the characteristic peritoneal spread of ovarian cancer [31]. Another point to emphasize is that we chose ESPEN criteria and hypoalbuminemia to define malnutrition as this was easily available in the NSQIP-PUF. Several validated tools ranging from screening questionnaires to biomarker-based assessments are available to evaluate malnutrition [32], yet no consensus exists regarding a gold-standard method for patients with ovarian cancer [33,34,35].

Pre-operative assessment of nutritional status in patients undergoing surgery for ovarian cancer should be incorporated in clinical practice, and included when counseling patients about potential operative complications. Further prospective research is needed to identify the optimal malnutrition assessment tool, to determine how malnutrition influences surgical outcomes when accounting for stage of disease and extent of surgery, and to evaluate whether pre-operative nutritional intervention can improve surgical outcome and decrease mortality.

## 5. Conclusions

In summary, in this cohort of patients undergoing surgery for ovarian cancer, malnutrition was associated with increased 30-day post-operative complications and mortality.

## Figures and Tables

**Table 1:** Baseline patients characteristics.

Variable	Malnutrition (N = 8744)	No Malnutrition (N = 11,430)	*p* Value
Age, years	58.6 ± 13.9	59.8 ± 13.0	<0.0001
BMI, kg/m^2^	28.7 ± 7.7	28.9 ± 7.1	0.061
Functional status prior to surgery:			0.0003
Independent	8594 (98.3%)	11,302 (98.9%)	
Partially dependant	111 (1.3%)	90 (0.8%)	
Totally dependant	15 (0.2%)	6 (0.1%)	
Unknown	24 (0.3%)	32 (0.3%)	
Diabetes (on medications)	915 (10.5%)	1316 (11.5%)	0.0185
Current smoking	1176 (13.5%)	1349 (11.8%)	0.0005
Hypertension	3107 (35.5%)	4405 (38.5%)	<0.0001
Pre-operative hematocrit	36.42 ± 5.3	36.97 ± 5.0	<0.0001
Pre-operative albumin	3.11 ± 0.5	4.08 ± 0.3	<0.0001
Frail ^a^	834 (9.5%)	1180 (10.3%)	0.065
Disseminated cancer ^b^	2325 (26.6%)	3840 (33.6%)	<0.0001
Ascites	1533 (17.5%)	1430 (12.5%)	<0.0001
ASA Classification ≥ 3	5184 (59.3%)	6603 (57.8%)	0.030

Continuous variables are presented as means ± standard deviation. Categorical variables are presented as n (%). ^a^ Calculated according to the modified 5-item frailty index. ^b^ Disseminated cancer is defined by NSQIP as cancer that has: (1) spread to one or more sites in addition to the primary site, AND (2) presence of multiple metastases indicating the cancer is widespread, fulminant, or near terminal. BMI—Body Mass Index; ASA—American Society of Anesthesiologists.

**Table 2:** Operative characteristics and post-operative discharge outcome.

Variable	Malnutrition (N = 8744)	No Malnutrition (N = 11,430)	*p* Value
Operative time, minutes	176.5 ± 93.2	194.1 ± 101.8	<0.0001
Days from operation to death ^a^	15.38 ± 8.5	11.09 ± 9.3	0.023
Total units of transfusion	2.47 ± 2.5	2.32 ± 3.6	0.424
Total length of hospital-stay, days	4.78 ± 4.7	4.17 ± 3.7	<0.0001
Days from operation to discharge	4.10 ± 3.9	3.82 ± 3.3	<0.0001
Discharge destination:			0.361
Home/Facility which was home	7241 (82.8%)	9409 (82.3%)	
Non-Home	1503 (17.2%)	2021 (17.7%)	

Continuous variables are presented as means ± standard deviation. Categorical variables are presented as n (%). ^a^ calculated for patients who died within 30-day post-operatively.

**Table 3:** Post-operative complications.

Variable	Malnutrition (N = 8744)	No Malnutrition (N = 11,430)	Odds Ratio	*p*-Value
Superficial SSI	286 (3.3%)	343 (3%)	1.09	0.274
Deep SSI	46 (0.5%)	38 (0.3%)	1.58	0.034
Organ/space SSI	241 (2.8%)	301 (2.6%)	1.04	0.591
Wound disruption	59 (0.7%)	71 (0.6%)	1.08	0.637
Pulmonary embolism	127 (1.4%)	128 (1.1%)	1.30	0.036
Deep vein thrombosis	105 (1.2%)	95 (0.8%)	1.45	0.008
Post-operative acute renal injury	20 (0.2%)	11 (0.1%)	2.37	0.017
Urinary tract infection	243 (2.8%)	333 (2.9%)	0.95	0.570
Sepsis	169 (1.9%)	169 (1.5%)	1.31	0.012
Septic shock	80 (0.9%)	61 (0.5%)	1.72	0.001
Pneumonia	168 (1.9%)	142 (1.2%)	1.55	0.0001
Re-Intubation	74 (0.9%)	46 (0.4%)	2.11	<0.0001
Cardiac arrest	21 (0.2%)	14 (0.1%)	1.96	0.046
Myocardial Infarction	60 (0.7%)	37 (0.3%)	2.12	0.0002
Stroke	19 (0.2%)	14 (0.1%)	1.77	0.098
Return to OR	220 (2.52%)	273 (2.4%)	1.05	0.560
Readmission	702 (8%)	781 (7%)	1.19	0.013
30-day mortality	73 (0.8%)	32 (0.3%)	2.99	<0.0001

Categorical variables are presented as n (%). *SSI*—Surgical Site Infection; *OR*—Operating Room.

## Data Availability

The data presented in this study are available on request from the corresponding author.
